# Global map of characterized dust sources using multisource remote sensing data

**DOI:** 10.1038/s41598-025-14794-3

**Published:** 2025-08-14

**Authors:** Ali Darvishi Boloorani, Masoud Soleimani, Ramin Papi, Nastaran Nasiri, Fatemeh Amiri, Najmeh Neysani Samany, Kan Huang, Iraj Gholami, Ali Al-Hemoud

**Affiliations:** 1 Department of Remote Sensing and GIS, Faculty of Geography, University of Tehran, Tehran, Iran; 2 Research Institute for Development of Space Science, Technology, and Applications, University of Tehran, Tehran, Iran; 3 GeoAI Environmental Engineering Consultancy L.L.C, Abu Dhabi, UAE; 4 National Cartographic Center (NCC), Tehran, Iran; 5 Center for Atmospheric Chemistry, Department of Environmental Science and Technology, Fudan University, Shanghai, China; 6 Division of Science, New York University Abu Dhabi, Abu Dhabi, UAE; 7 Environment and Life Sciences Research Center, Kuwait Institute for Scientific Research, Kuwait City, Kuwait

**Keywords:** Dust source mapping, Remote sensing, Sentinel-5P, Absorbing aerosol index (AAI), Environmental sciences, Hydrology, Natural hazards, Planetary science, Engineering

## Abstract

The most recent high-resolution global map of dust emission sources is provided by Ginoux et al. (2012), which utilizes an aerosol loading approach based on time series of MODIS Aerosol Optical Depth (AOD). However, advancements in remote sensing technology and analytical techniques have created a growing need for more accurate and up-to-date maps of global dust sources to enhance the understanding and management of this phenomenon. In this study, we first calculated the global mean Sentinel-5P Absorbing Aerosol Index (AAI) for the period 2018–2024. Regions with AAI values greater than 0.25 were identified as potential dust sources through histogram analysis validated by ground truth data. Next, areas without dust emission potential were excluded from the mean AAI map using a multi-stage masking process that considers land surface characteristics such as soil depth, permanent water bodies, and built-up areas. Validation results demonstrate strong performance, with a Precision of 84.7%, Recall of 80.7%, and F1-score of 82.6%, confirming the reliability of the global dust source map produced. The findings indicate that about 5% of the world’s land area acts as a dust emission source, mainly located in North Africa (67%) and Asia (30%). Land use/land cover analysis reveals that global dust sources comprise deserts, vegetative, and hydrological categories, accounting for 65%, 26%, and 9%, respectively. Among these, sandy areas, rangelands, and intermittent water bodies exhibit the largest extent on a global scale, respectively. Natural and human factors contribute 65% and 35%, respectively, to the formation of global dust sources. The frequency of dust events from desert sources has experienced an increasing trend worldwide, but in the case of non-desert sources, it has decreased in some regions, such as the Middle East. This study focused on identifying major dust emission sources based on relatively high aerosol loads over time. Our results provide a new global dust atlas that can serve as a practical foundation for climate modeling and for formulating disaster risk reduction and management plans.

## Introduction

In recent decades, land use/land cover (LULC) change, desertification, and deforestation affected by climate change and prolonged droughts^1^ caused by anthropogenic and natural processes^2–6^ have led to the expansion of dust sources, particularly in arid and semi-arid regions, such as the Middle East. Dust storms impact various Earth components, such as cloud nucleation and precipitation processes, radiative forcing, hydrological cycles, terrestrial and marine biogeochemical cycles, vegetation health and productivity, water pollution, soil fertility and food production, etc., and pose a significant threat to human health^7–10^. Identifying dust emission sources is the first necessary step in managing this phenomenon and reducing/controlling its adverse effects^11^. In addition, this is important for understanding the environmental and climatic mechanisms for the development of global dust forecasting and climate models^12–14^.

Dust storms and their various consequences are not limited to the emission sources; fine-grained dust particles can be carried by wind thousands of kilometers across the hemisphere^15^, affecting even pristine remote areas and the polar regions^16^. Hence, dust is considered a global hazard because it mainly arises from the interaction of environmental and human factors on a regional or global scale^17^, and can impact anywhere as a function of the general atmospheric circulation. Accordingly, dust-related studies, especially in terms of identifying emission sources and impact areas, seem to require a global perspective, independent of natural and political boundaries.

Due to its critical importance, the topic of dust source identification/mapping has been studied extensively on a global scale^12,18–26^. Given the constraints of ground-based/field observations^27^, most research relies on satellite remote sensing data^28^. Dust storms are very dynamic and short-lived phenomena that can disperse quickly after emergence. Hence, the remote sensing sensor must have high temporal and spatial resolutions to monitor dust events effectively^29,30^.

According to the literature, the most employed approaches to identify and characterize dust emission sources that take advantage of various satellite remote sensing data fall into three main categories: expert knowledge-based visual interpretation^31,32^, simulation model-based^12,20,22^, and aerosol load-based^25,26^. Each approach has its strengths and limitations, and since they are suited for different applications, determining which method is superior can be challenging. Visual interpretation focuses more on accurately identifying hotspot dust sources at typically local scales, as its implementation on a large scale is very costly and time-consuming. Moreover, this approach is inherently subjective, relying heavily on expert knowledge. Consequently, if the interpreter lacks sufficient experience and skills in interpreting satellite imagery, the level of uncertainty can increase significantly^33^. Model-based approaches often focus on the large-scale simulation of emission and deposition rates, their characteristics, and variability across different dust emission sources and impact areas^34^. Aerosol load-based approaches are centered on analyzing the long-term spatial-temporal behavior of ambient columnar atmospheric aerosol loading observed through satellite imagery^33^.

The latter approach, despite its shortcomings, particularly the uncertainty of satellite aerosol products, is more cost-effective and applicable in terms of processing cost and time to identify and characterize dust sources on a global scale. In this regard, the Moderate Resolution Imaging Spectroradiometer - Aerosol Optical Depth (MODIS-AOD), include the “deep-blue”^35,36^ and “dark-target”^37^ algorithms developed to obtain the aerosol optical thickness over bright land areas and oceans and predominantly vegetated (dark) land surfaces, is considered the most widely used satellite aerosol product for dust studies^26,38–40^, and due to its fine nominal spatial resolution of 3–10 km and sub-daily temporal resolution (two acquisitions per day by Terra and Aqua with equatorial overpass times of 10:30 and 13:30, respectively), near-global spatial coverage, relatively long temporal coverage (from 2000 to the present), wavelength diversity (0.412, 0.47, 0.65, and 2.13 μm), free availability^33^, and relatively high correlation with the ground-based AERONET-retrieved AOD data^41,42^. However, due to the limitations of existing datasets, such as MODIS-AOD, which is subject to high uncertainty influenced by aerosol type, elevation, and LULC^43,44^, as well as given the rapid advancements in satellite remote sensing technologies and algorithms, there remains a pressing need to explore and utilize new remote sensing data and methodologies.

To the best of our knowledge, one of the most comprehensive maps of global dust sources has been produced using aerosol load approaches based on MODIS-AOD by Ginoux et al. (2012)^26^. Uncertainties in MODIS-AOD retrievals, limitations in spatial resolution, potential overestimation of AOD values due to dust transported from upwind sources, and the omission of variability in dust emission potential among different LULC classes may introduce errors that substantially undermine the map’s reliability^11,39^. In this context, the sun-synchronous Sentinel-5P satellite–the European Space Agency’s (ESA) first Copernicus mission dedicated to high spatial and temporal resolution atmospheric measurements of air quality, ozone and ultraviolet (UV) radiation, and climate monitoring–has now provided over seven years of data. This presents valuable opportunities to leverage its products for identifying global dust sources and addressing the limitations of previous datasets.

The Absorbing Aerosol Index (AAI) is an atmospheric parameter measured by the TROPOMI instrument onboard the Sentinel-5P satellite. It is derived from wavelength-dependent variations in Rayleigh scattering within the UV spectral range, specifically at two wavelengths: 354 and 388 nm. The AAI is calculated by subtracting the observed reflectance captured by the sensor from the modeled reflectance, which represents the theoretical reflectance of the atmosphere during Rayleigh scattering. This index illustrates the concentration of UV-absorbing aerosols in the atmosphere, such as dust particles and smoke. Since ozone absorption is minimal at these two wavelengths, the Sentinel-5P AAI is less affected by atmospheric cloud cover compared to the MODIS-AOD measurements^45^.

Our study aims to identify global dust sources by developing a novel aerosol load-based approach that leverages the unique capabilities of the Sentinel 5P-AAI time series data. Given the key role of LULC in dust formation, we employ a multi-stage masking approach based on histogram analysis using land surface characteristics (e.g., soil depth, permanent water bodies and built-up areas) to minimize the potential influence of false AAI values on the identification of dust emission sources. Since various environmental factors—such as desert aeolian processes, vegetation dynamics, and hydrological conditions—contribute to the formation of dust storms, the identified sources are ultimately characterized by their origin and type, based on the dominant driving factors. This research presents a global dust atlas that offers critical insights for policymakers worldwide, enabling the development of more effective adaptation and mitigation strategies to address the impacts of dust storms.

## Methodology

### Identification of potential dust sources

In this study, areas exhibiting consistently high atmospheric aerosol loads over time are identified as potential dust emission sources. The time series of the daily Sentinel 5P-AAI product with a spatial resolution of 1 km from 2018 to 2024 is employed to analyze the spatiotemporal patterns of ambient atmospheric aerosol loading. The AAI is a dimensionless measure of the columnar prevalence of aerosols in the atmosphere. It is derived from the spectral contrast between a pair of UV wavelengths, where the difference between the observed and modeled reflectance results in a residual. A positive residual indicates the presence of UV-absorbing aerosols, such as dust and smoke. Clouds typically yield residuals near zero, while strongly negative residuals may suggest the presence of non-absorbing aerosols, including sulfate aerosols.

To this end, AAI’s daily global products (OFFL/L3_AER_AI) are first averaged over the period using the Google Earth Engine (GEE) data catalog. Accordingly, areas with higher positive AAI values can be interpreted as potential dust sources. However, the choice of a specific AAI threshold significantly influences the spatial pattern and boundary of detectable potential dust sources. According to the approach proposed by Darvishi Boloorani et al. (2023c), the primary dust-emitting areas in the Middle East are initially identified as ground truth data through the visual interpretation of sub-daily true-color composite MODIS-Terra & Aqua imagery for the period from 2018 to 2024^33^. This approach relies on the human analyst context-based subjective judgment. The Middle East has a variety of dust emission sources, including desert, vegetative, and hydrological^46^, which can be a good representative of other dust source regions, taking into account the necessity for minimum processing and time costs associated with the visual interpretation approach. Ground truth dust areas are identified using the visualization capabilities of NASA’s EOSDIS web-based application and the on-screen digitizing method using ArcGIS 10.8 tools. These areas are correspond to landforms that have experienced dust emission hotspots at various times due to the interaction of different drivers such as high temperatures, high evaporation rates, low soil moisture content, sparse or poor vegetation coverage, fine-grained eroded soil particles, and erosive winds^47^. Hotspots are defined as locations in source areas where the highest emission rates are observed during dust events^17^. Accordingly, ground truth dust sources are areas where several dust emission hotspots are observed over time on MODIS true-color composite images as a result of hydroclimatic dynamics. During the visual interpretation process, the boundaries of ground truth dust sources are determined by considering the characteristics of landforms and prevailing wind directions in areas associated with dust emission hotspots.

**Fig. 1:**
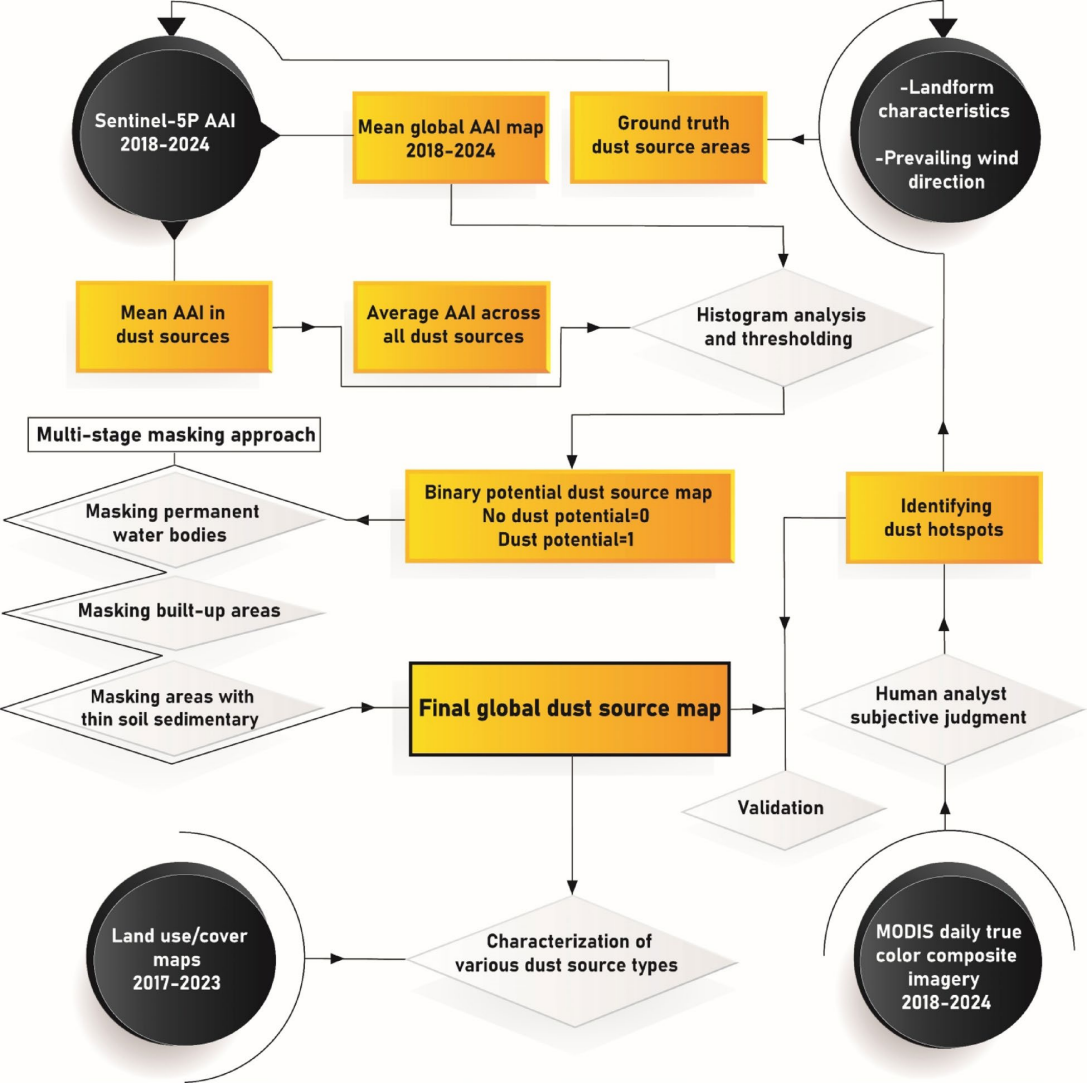
Proposed approach to identify and characterize global dust sources using multi-source satellite remote sensing data.

Next, the mean AAI values associated with pixels within the ground truth dust areas during the period 2018–2024 are extracted. Finally, the mean AAI across all identified dust areas in the Middle East is calculated and considered as a general threshold for use in a histogram slicing process. This approach enables classifying the global mean AAI product into two binary categories: 0, indicating no potential dust source, and 1, indicating a potential dust source. This procedure facilitates the determination of the optimal AAI threshold for identifying potential dust sources on a global scale. Fig. 1 presents a schematic overview of this stepwise methodology.

### Dust source mapping

Due to the common uncertainties associated with aerosol load-based approaches to dust source identification, it is necessary to consider the spatiotemporal behavior of land surface characteristics to minimize overestimation and refine the potential dust source maps^39^. Accordingly, false AAI-based columnar aerosol concentration values due to wind direction and topography patterns are largely excluded by eliminating areas without dust emission potential. This allows for the transition from potential dust sources to dust sources. In fact, in this process, the behavior of the Earth’s surface characteristics is linked to the columnar atmospheric aerosol, which referred to as “dust source mapping”.

Geology, pedology, soil moisture, and LULC are the primary surface characteristics that play a role in the formation of dust sources. According to the literature, many other factors can be effective, but they are often correlated with these four characteristics^17,48^. Here, a multi-stage masking approach is utilized to consider soil thickness, water bodies, and built-up areas, which helps eliminate falsely detected dust sources. Binary masks (no dust emission potential = 0, dust emission potential = 1) are created from each of these parameters and then multiplied by the mean AAI product. Finally, the regions remaining after these steps are labelled as dust sources (Fig. 1). The specifications of the binary masks are presented in Table 1.

**Table 1:** Data used and details of the multi-stage masking approach.

Parameter	Data source	Spatial resolution	Time span	References	Description of produced mask
Soil thickness	Soil and sedimentary deposit thickness	1 Km	1900–2015	^49^	Areas with thin sedimentary soil layers, particularly those with a thickness of less than 5 m, are unlikely to produce dust storms, even if other conditions are favorable. Therefore, regions with soil depths below this threshold are excluded from potential dust source considerations
Water bodies	JRC Yearly Water Classification History, v1.4- GEE data catalog	30 m	1984–2021	^50^	Since water bodies do not emit dust–mainly because moisture causes soil particles to stick together–the maximum extent of global permanent inland water bodies is designated as areas with no dust emission potential
Built-up areas	Esri Land Cover	10 m	2017–2023	^51^	Built-up areas and impervious urban surfaces do not contribute to dust emissions. To account for spatiotemporal changes in LULC, built-up areas persisting for at least one year between 2017 and 2023 are classified as having no dust emission potential

### Validation of the dust source map

Standard binary classification metrics, including precision, recall, and F1-score, are employed to evaluate the spatial accuracy of the generated global dust source map. The evaluation is conducted by comparing the binary dust source outputs produced by the proposed methodology with ground truth dust and non-dust hotspots identified through visual interpretation of MODIS true-color composite imagery across the Middle East. Non-dust hotspots are carefully selected from regions with no observed dust emission potential during the 2018–2024 period, including areas such as dense vegetation, water bodies, urban environments, rocky mountains, and other naturally stable surfaces.

To compute the validation metrics, a confusion matrix (Table 2) is constructed by comparing the classified outputs of the dust source map with the corresponding ground truth classes derived from visual interpretation. The matrix summarizes classification outcomes using four standard metrics: True Positives (TP), False Positives (FP), True Negatives (TN), and False Negatives (FN)^52,53^. Here, TP refers to the number of dust hotspots that truly fall within identified dust sources. FP represents the number of dust hotspots that falsely fall in non-dust sources (Type I error). TN is the number of non-dust hotspots that truly fall within non-dust sources. FN denotes the number of non-dust hotspots that falsely fall in dust sources (Type II error). These metrics form the basis for calculating precision, recall, and the F1-score to quantitatively evaluate the spatial accuracy of the dust source map through Eqs. (1)–(3)^53^.

**Table 2:** Confusion matrix for validation of proposed methodology.

		Ground truth class	
		Dust hotspot (1)	Non-dust hotspot (0)
Map class	Dust source (1)	a	b
	Non-dust source (0)	c	d

1 $$Precision = \frac{{TP}}{{TP + FP}}$$

2 $$Recall\left( {Sensitivity} \right) = \frac{{TP}}{{TP + FN}}$$

3 $$F1\;score = 2 \times \frac{{\left( {Precision \times Recall} \right)}}{{\left( {Precision + Recall} \right)}}$$

where according to Table 2, $TP = a/\left( {a + b} \right)$, $FP = b/\left( {b + d} \right)$, $TN = d/\left( {c + d} \right)$, and $FN = c/\left( {a + c} \right)$.

### Categorization of various dust sources

Given that dust storm formation is influenced by multiple environmental factors, the emission sources can differ in their origins^46^. By incorporating various LULC classes and landforms with potential for dust emission into the global dust source map, we can discriminate between different types of dust sources (Fig. 1). This approach ensures a comprehensive understanding of dust source typologies, facilitating targeted analysis and mitigation strategies.

This study utilizes the Esri Land Cover, Global Lakes and Wetlands Database (GLWD), and OpenLandMap^54^ datasets, combined using geometric-based intersection functions in ArcGIS 10.8 to separate dust sources associated with vegetation (cropland and rangeland), hydrology (lake, intermediate water, floodplain, wetland, coastal, and swamp forest), and desert.

To differentiate cropland and rangeland dust sources, areas consistently classified under these categories for at least three years between 2017 and 2023 are selected, accounting for spatial-temporal variability and potential classification errors in the Esri Land Cover product. Next, “vegetative dust sources” are defined as areas of global dust sources that overlap with relatively persistent classes of cropland and rangeland. “Vegetative dust sources” are then defined as regions within the global dust source map that intersect with stable cropland and rangeland classifications. To separate coastal areas as one of the “hydrological dust sources”, the Digital Elevation Model (DEM) from the Shuttle Radar Topography Mission (SRTM) (Farr et al., 2007) with a spatial resolution of 30 m is used. To delineate coastal zones as a subset of “hydrological dust sources,” the Digital Elevation Model (DEM) from the Shuttle Radar Topography Mission (SRTM) (Farr et al., 2007), with a spatial resolution of 30 m, is employed. Accordingly, coastal dust sources are defined as regions within approximately 5 km of oceans and seas with an elevation of less than 20 m above sea level. Coastal dust sources are defined as areas located within approximately 5 km of oceanic or sea boundaries and exhibiting elevations below 20 m above sea level. After completion of the above separation and labeling steps, the remaining areas are considered to be “desert dust sources”. Following the above classification and labeling procedures, residual regions are categorized as “desert dust sources.” Desert dust sources are further categorized into “sandy” and "non-sandy" sources by incorporating detailed classifications of different soil types. These desert dust sources are further subdivided into “sandy” and “non-sandy” categories based on detailed soil type classifications. To discriminate between cropland and rangeland dust sources, due to spatial-temporal variations and uncertainties associated with image classification errors in the Esri Land Cover product, areas that have been in these classes for at least three years between 2017 and 2023 are considered. Next, “vegetative dust sources” are defined as areas of global dust sources that overlap with relatively persistent classes of cropland and rangeland. To separate coastal areas as one of the “hydrological dust sources”, the Digital Elevation Model (DEM) from the Shuttle Radar Topography Mission (SRTM)^55^ with a spatial resolution of 30 m is used. Accordingly, coastal dust sources are defined as regions within approximately 5 km of oceans and seas with an elevation of less than 20 m above sea level. After completion of the above separation and labeling steps, the remaining areas are considered to be “desert dust sources”. Desert dust sources are further categorized into “sandy” and “non-sandy” sources by incorporating detailed classifications of different soil types.

### Analysis of dust spatial-temporal patterns

To derive a global dust spatial-temporal profile, the month exhibiting the highest AAI value is determined for each pixel. This analysis enables identification of peak dust activity periods across different global regions. A similar approach is performed to determine the year with maximum dust activity, using daily MODIS-AOD due to the long-term data availability (2003–2023).

Additionally, we performed a change detection analysis (Eq. 4) for the MODIS-AOD on a global scale by comparing the mean AOD values between two periods: 2003–2013 and 2013–2024. Conventional break/change point detection analysis methods, including von Neumann’s ratio test^56^, Pettitt’s test^57^, standard normal homogeneity test (SNHT)^58,59^, and Buishand’s test^60,61^ are applied to determine the turning point in the yearly AOD time series. Both Pettitt’s test and SNHT yielded consistent results, indicating that 2013 represents a statistically significant turning point (P-value < 0.05).

4 $$Change\;detection = \frac{{AOD_{{2013 - 2024}} - AOD_{{2003 - 2013}} }}{{AOD_{{2003 - 2013}} }} \times 100$$

This approach provided a more robust understanding of spatial dust emission trends over a longer period, complementing the shorter-term insights provided by the AAI data. The integration of these datasets provided a clearer picture of both current and historical trends in dust activity, enabling more informed conclusions and strategies for dust management.

## Results and discussion

### AAI-based aerosol load approach

This study employs a novel global dust source identification/mapping approach based on aerosol loading and time series of the Sentinel 5P-AAI daily products. This approach incorporates the spatial-temporal behavior of AAI within ground truth dust areas to accurately identify global dust sources. Previous studies have utilized long-term mean aerosol load satellite products, such as MODIS-AOD, for dust source identification^62^. However, since AOD values at a given pixel can be influenced by dust transported from upwind sources, relying solely on AOD for identifying dust emission sources is limited in effectiveness and subject to considerable uncertainty^63^. Consequently, applying thresholds to aerosol load products offers a practical and operationally feasible solution for reducing potential errors and improving the accuracy of dust source identification^39^.

**Fig. 2:**
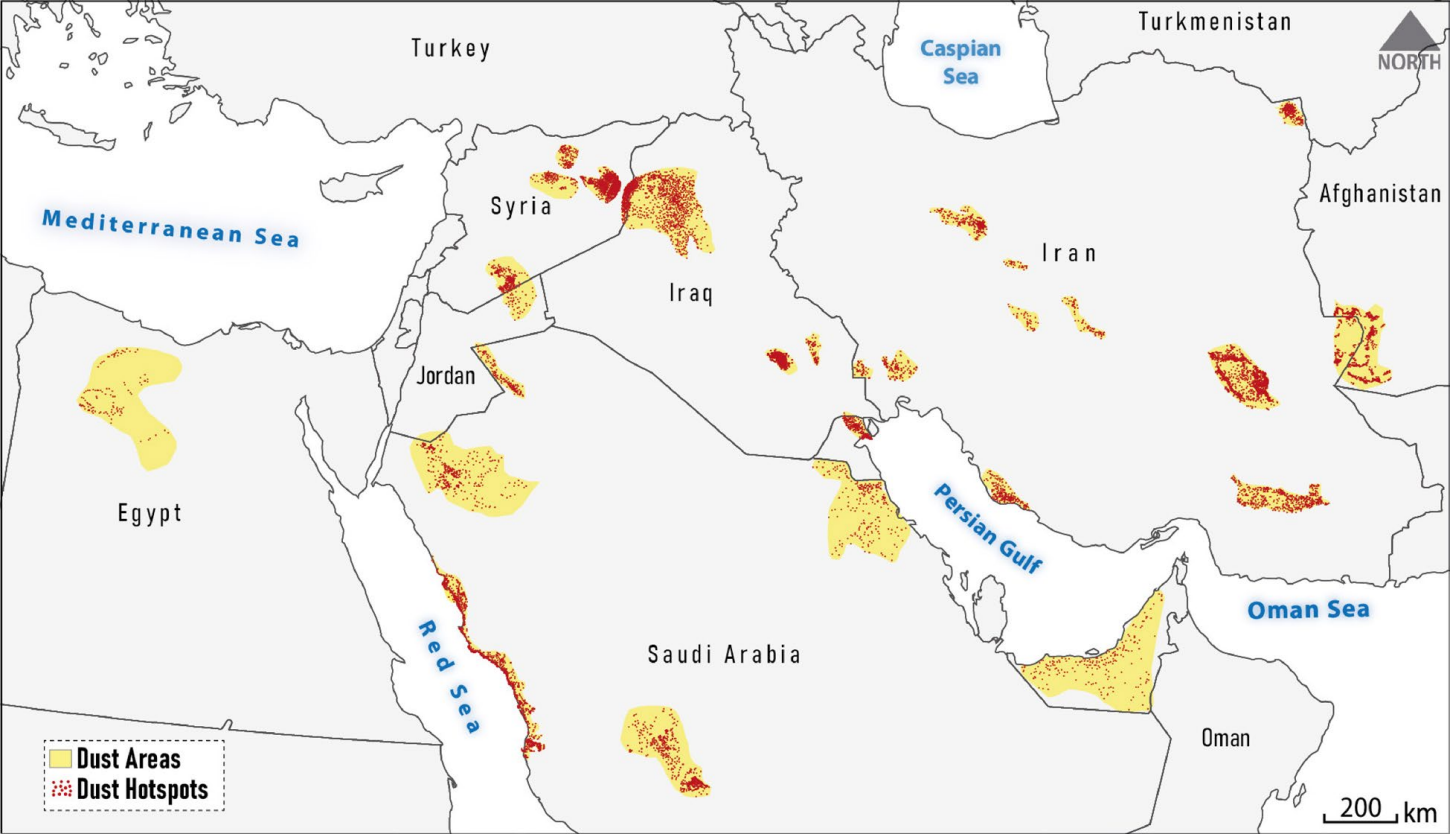
Ground-truth main dust areas and associated dust emission hotspots in the Middle East identified by visual interpretation of MODIS-Terra & Aqua sub-daily true color composite imagery (2018–2024) to Sentinel 5P-AAI thresholding process.

Here, the mean AAI of the main dust areas in the Middle East for 2018–2024 was considered as the basis for determining the appropriate threshold. These main dust areas (comprising a total of 26 landform-related polygons) were identified through visual interpretation of sub-daily MODIS true-color composite imagery as ground truth data (Fig. 2). During the visual interpretation process, a total of 11,242 dust emission hotspots were observed in parts of these polygons as a function of hydroclimatic conditions^11^ from 2018 to 2024. Since the spatiotemporal pattern of AAI is a function of wind speed and direction, values in downwind pixels are usually highly correlated with upwind areas. Therefore, to achieve a generalizable threshold, instead of extracting pixel-wise AAI values in dust emission hotspots, we considered the spatial neighborhood behavior by calculating the mean AAI across polygons of dust areas (Fig. 2). Mean AAI values in these polygons range from about 0.02 (in central Syria) to 1.11 (in Saudi Arabia). The average AAI across all polygons is 0.25, which was applied as a threshold to classify the global mean AAI map into non-potential dust sources (labeled as a binary value of 0) and potential dust sources (labeled as a binary value of 1).

### Distribution of global dust sources

By excluding areas with no dust potential (e.g., thin soil thickness, permanent inland water bodies, and built-up areas) from the binary classified global mean AAI map (considered as potential dust sources), a relatively accurate boundary map of global dust sources was extracted. Our results indicate that, global dust sources are mainly located in the Earth’s desert belt. The spatial distribution of global dust sources, with a total area of 7,892,663 km^2^, across continents shows that Africa is the largest contributor with an area of 5,301,025 km^2^ (67.16%). Asia comes second with 2,395,173 km^2^ (30.34%), reflecting the continent’s large arid and semi-arid regions, particularly in the Middle East, Central Asia, and East Asia. The remaining 2.5% of global dust sources are located in North America (Southwestern United States) with an area of 85,552 km^2^ (1.1%), South America (Northern Argentina) with an area of 56,489 km^2^ (0.71%), Australia with an area of 54,281 km^2^ (0.68%) and Europe (only in Spain) with an area of 143 km^2^ (0.001%).

**Fig. 3:**
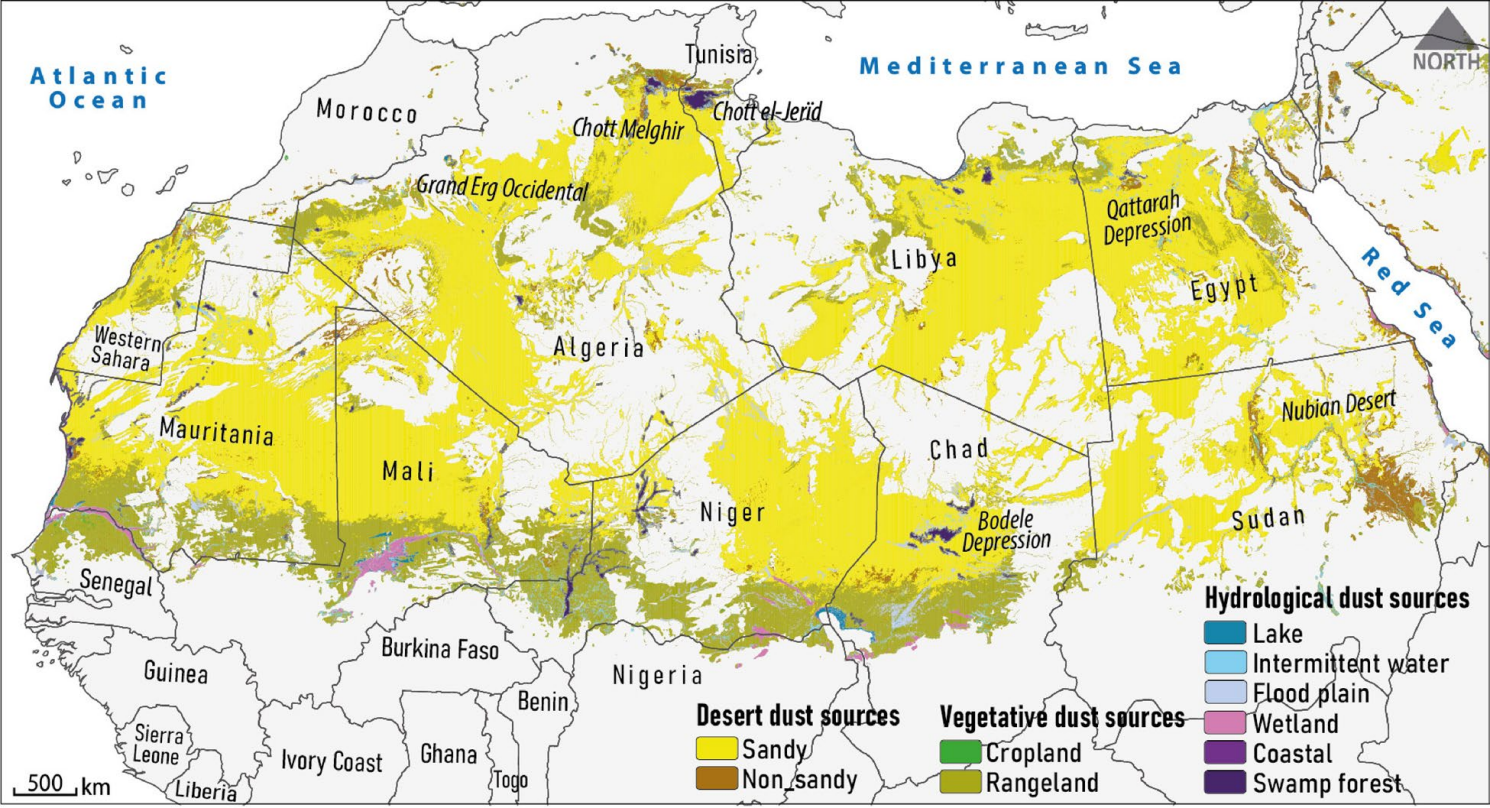
Distribution of various dust source types in the North Africa region.

**Fig. 4:**
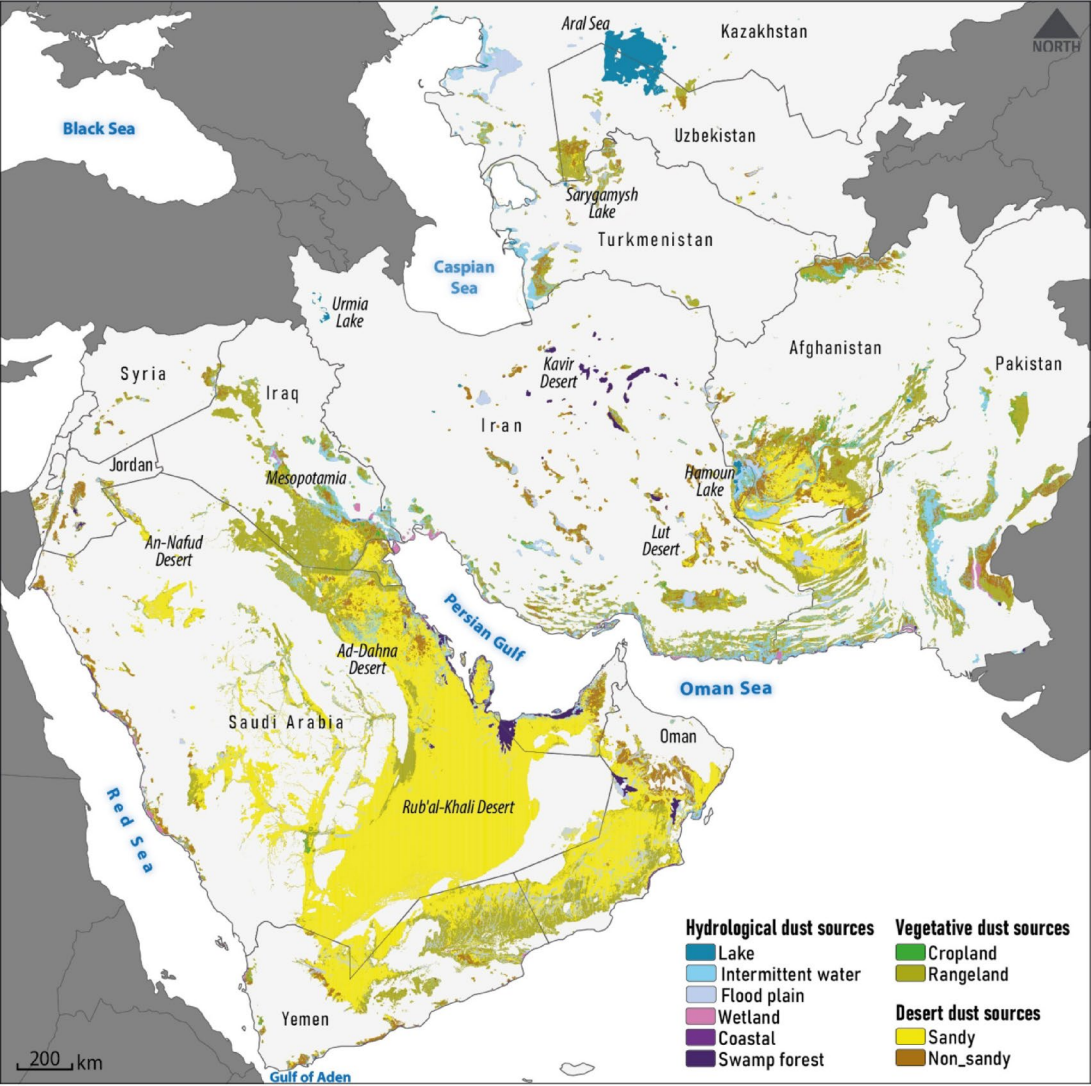
Distribution of various dust source types in the Southwest and Central Asia region.

**Fig. 5:**
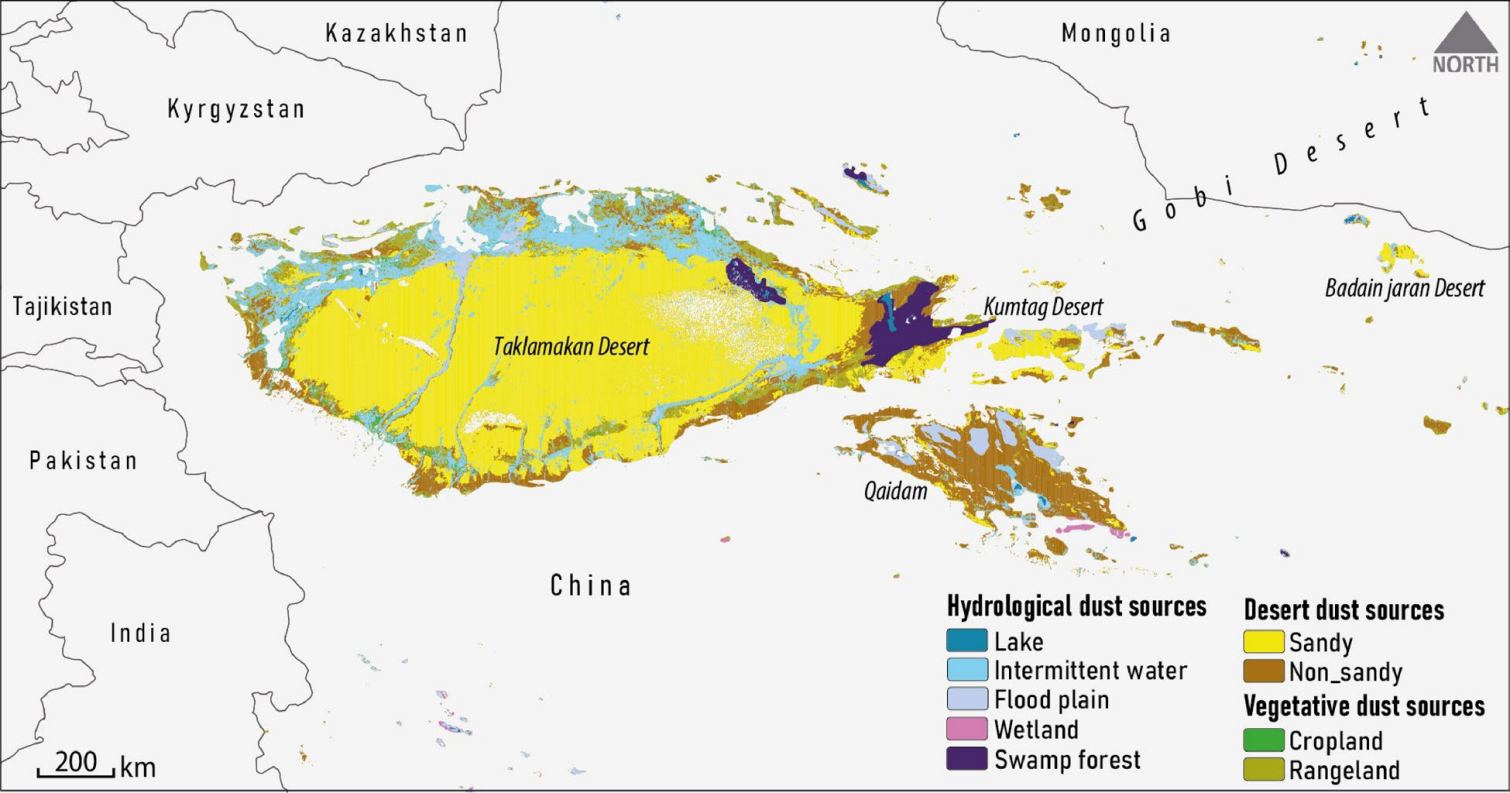
Distribution of various dust source types in the East Asia region.

According to the LULC analysis, global dust sources can be broadly classified into three categories: desert ($\:\sim$65%), vegetative ($\:\sim$26%), and hydrological ($\:\sim$9%). Given that 97.5% of identified global dust sources are located in Africa and Asia, this study specifically focuses on describing the spatial distribution of dust sources within these continents. Dust sources on these continents are mainly located in the regions of North Africa, Southwest Asia, Central Asia, and East Asia. To illustrate this distribution, separate maps of dust sources are presented for North Africa (Fig. 3), Southwest and Central Asia (Fig. 4), and East Asia (Fig. 5).

#### North Africa dust sources

As shown in Fig. 3, dust sources in Africa are primarily located in the northern region, particularly in the vast Sahara, the largest hot desert on Earth. North Africa is a major contributor to global dust emissions due to its extremely dry climate, sparse vegetation, and extensive sandy soils^64^, combined with anthropogenic pressures such as overgrazing and deforestation^65^.

Our results show that, about 70% of the dust sources in this region are desert-type. The vegetative and hydrological sources of dust storms are mostly located in the southern latitudes of the North Africa region, closer to the equator, where the climate is more humid but still experiences seasonal dry periods^12^. Dust storms in these areas, such as the Sahel and near the Sahara, originate from dried beds of lakes, seasonal wetlands, and floodplains. During the dry season, as these water bodies shrink, their fine-grained fluvial and alluvial sediments are eroded by aeolian processes. Seasonal variations invegetation cover also contribute to dust emissions^12^.

Vegetative dust sources are often located on the marginal lands surrounding deserts, which account for about 25.5% of Africa’s sources. In addition, over 98% of the area of ​​vegetative dust sources is associated with rangelands. Regarding hydrological dust sources, which account for 4.5% of the area of the North African dust sources, the largest share is from flood plains (33%), swamp forests (24%), intermittent waters (20%), and wetlands (18%), respectively.

The dominant trade winds, particularly the Harmattan and Sirocco, which cross the Mediterranean, play an important role in the emission and transport of dust from the North African region^66,67^. These winds often carry dust particles over long distances, sometimes reaching the United States^68^ and even East Asia^69^.

#### Southwest and central Asia dust sources

Most dust sources in the Southwest and Central Asia regions are found in deserts ($\:\sim$53%), particularly in sandy deserts ($\:\sim$87%) such as the An-Nafud, Ad-Dahna, and Rub’ al-Khali deserts in Saudi Arabia, as well as large parts of Oman and Yemen (Fig. 4). These deserts are primarily natural dust emission sources, driven by their hot and dry climate and unconsolidated sand particles that lead to frequent regional dust events^70,71^. Vegetative dust sources in these regions account for a significant share of 33%, of which 94% is associated with rangelands. Vegetative dust sources are mainly located in the Tigris and Euphrates river basins, the Helmand river basin in Afghanistan, and the Indus river basin in Pakistan. The presence of ephemeral and dried-up lakes and wetlands in the Mesopotamian region of Iraq, Central Iran, the Sistan Plain (Hamoun Lakes) on the Iran-Afghanistan border, and the Aral Sea on the Kazakhstan-Uzbekistan border has given Southwest and Central Asia a higher share of hydrological dust sources ($\:\sim$14%) compared to the North Africa region. Intermittent waters constitute the largest share ($\:\sim$38%) among the various types of hydrological dust sources, covering an area of over 97,000 km^2^. The share of floodplain-related dust sources ($\:\sim$34%) is also important in the second degree. In general, three main wind systems, including the Shamal winds over the Arabian Peninsula, the northerly Levar winds (wind of 120 days) over southwest Asia, and the low-level southwesterly monsoon jet blowing over the western Arabian Sea, characterize dust activity in this region^62^. These winds interact with other hydroclimatic and environmental conditions to cause dust emissions from sources at different times of the year, from spring to winter. However, dust emissions in this region are often caused by the Shamal and Levar wind systems, which are active during spring and summer^72,73^.

#### East Asia dust sources

The spatial distribution of different dust source types in the East Asian region is shown in Fig. 5. As evident, the largest area ($\:\sim$ 69%) is associated with desert dust sources, which also have a higher share compared to sources in North Africa and Southwest and Central Asia. The primary sources of dust emissions in East Asia are large desert areas, particularly sandy deserts such as the Taklamakan, Badain Jaran, and Kumtag. Classified as cold deserts, these regions are primarily located in China, which lies in the rain shadow of the Tibetan Plateau and other Central Asian highlands (e.g., the Himalayas), resulting in a cold and dry climate^74^. These cold deserts contribute significantly to the occurrence of dust storms in the region. The Taklamakan Desert, known for its vast sand dunes and extremely dry conditions^75^, is a major natural source of dust emissions^76^.

East Asia exhibits the lowest proportion of vegetative dust sources, accounting for approximately 13%, with rangelands playing a dominant role, representing about 89% of vegetative sources compared to the other regions. Conversely, hydrological dust sources are more prominent in this region, constituting approximately 18%. Within this hydrological category, intermittent water bodies cover the largest area, comprising 56% of the hydrological dust sources. Although less extensive than desert regions, hydrological sources nonetheless have a significant influence on the overall dust dynamics in East Asia^77,78^.

In this region, dust storms primarily result from near-surface wind regimes that are closely tied to the broader atmospheric circulation patterns and the local topography. Two prevailing wind directions play a role in the formation of dust storms in the region, particularly in the Taklamakan Desert. Generally, the eastern parts of the desert are affected by northeasterly winds, while the western parts experience northwesterly winds. Additionally, the southwestern edge of the desert experiences the highest frequency of dust storms, driven by persistent northwesterly winds^79^.

### Characterization of global dust sources

**Fig. 6:**
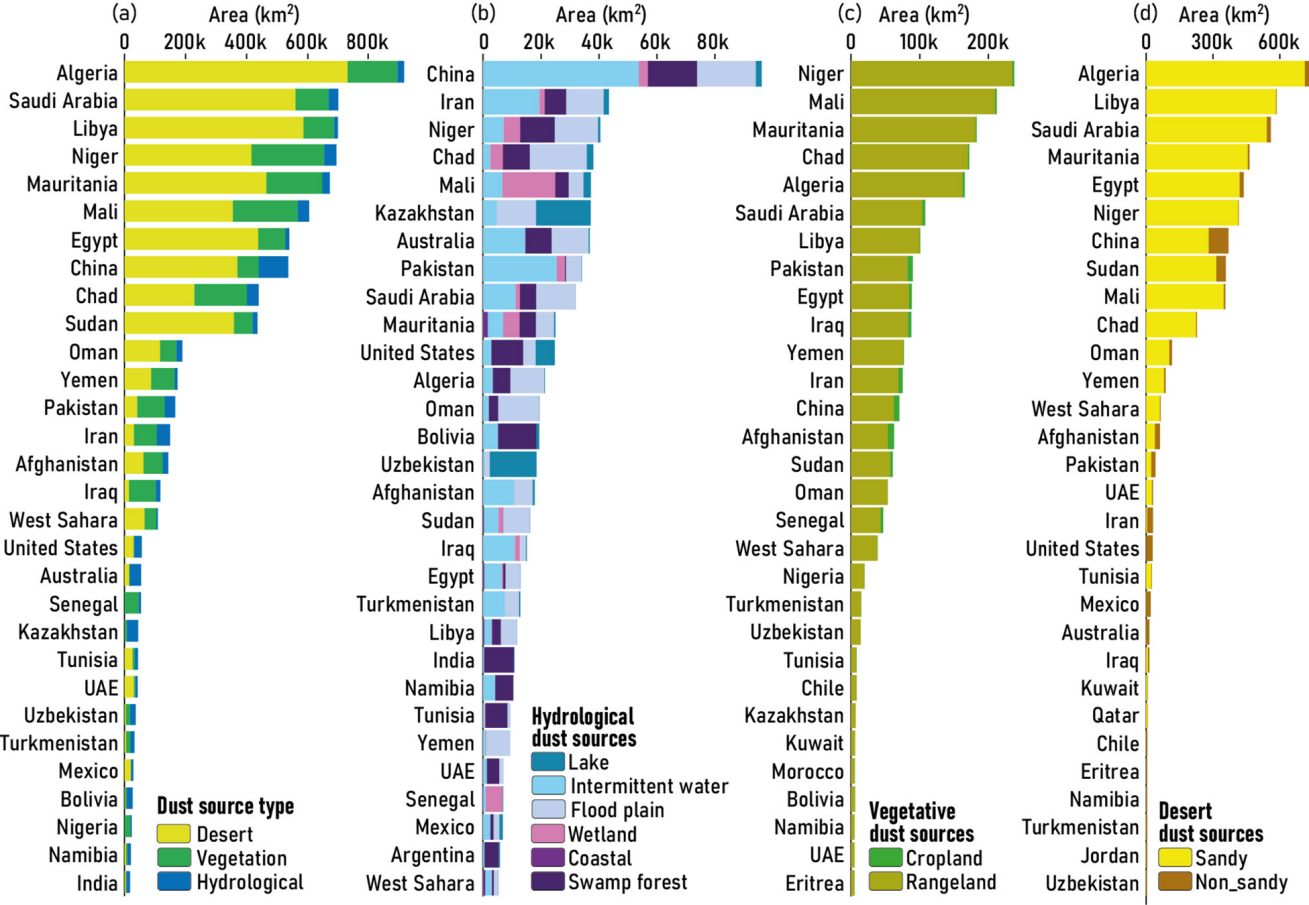
Distribution of various types of dust sources across the global involved countries: (**a**) primary categories of dust sources, including desert, vegetative, and hydrological; (**b**) hydrological dust sources; (**c**) vegetative dust sources; and (**d**) desert dust sources.

Fig. 6a shows the global distribution of the area of various dust source types in the involved countries. Accordingly, Algeria, with an area of more than 900,000 km^2^, contains the largest share ($\:\sim$12%) of global dust sources, including desert (79.63%), vegetative (18.05%), and hydrological (2.32%). In contrast, India has the smallest share (18,000 km^2^) of global dust sources, of which approximately 60% are hydrological. In most of the countries involved, except Pakistan, Iran, Iraq, and Australia, desert dust sources account for the largest share. On the other hand, no desert dust sources have been identified in some of the involved countries, including Senegal, Kazakhstan, Uzbekistan, Turkmenistan, Bolivia, Nigeria, Namibia, and India. In the second rank, vegetative dust sources also cover a significant area in many countries involved. Fig. 6b, c and d show the area distribution of various types of hydrological, vegetative, and desert dust sources, respectively.

Hydrologically, China has the largest share of dust sources, with over 96,000 km^2^, of which about 56% are associated with dried beds of intermittent waters. After China, Iran ranks second in terms of the extent of hydrological dust sources, with a reduction of about 46%. In Iran, similar to China, the largest share of hydrological dust sources is associated with intermittent waters ($\:\sim$ 45%). Meanwhile, flood plains also represent a significant contribution to the hydrological dust sources in most of the countries involved (Fig. 6b). As pointed out in previous studies, the formation of hydrological dust sources such as lakes, intermittent waters, flood plains, and wetlands in countries located in the Middle East and North Africa (MENA) region is due to water crises resulting from factors such as reduced precipitation, increased temperature, periodic droughts, population growth, agricultural expansion, and unsustainable water resource management^46,80^. In this regard, many studies have highlighted the role of dried-up water bodies (wetlands, lakes, and rivers), particularly in the Mesopotamian Marshes in Iraq^29^, Urmia Lake^81,82^ and the Hamoun Lakes^83,84^ in Iran, and the Aral Sea between Kazakhstan in the north and Uzbekistan in the south^85,86^, in dust emissions and their widespread impacts.

In terms of vegetative dust sources, Niger is the largest contributor with an area of over 237,000 km^2^, of which approximately 99% is associated with rangeland. In the other 29 countries involved, the highest share of dust sources came from rangelands (Fig. 6c). It should be noted that the low share of cropland as a source of dust storms does not necessarily mean that rangelands could always contribute more to global dust emissions. The reason for our finding is largely dependent on LULC^51^, as the area of rangelands is much larger than that of croplands in all countries. Countries located in desert areas with very limited agricultural activities, such as Niger, Mali, Mauritania, Chad, Algeria, Saudi Arabia, etc., have a very low potential for the formation of cropland dust sources. To better understand this issue, in countries such as China, Afghanistan, Pakistan, and Iran, which have more extensive agricultural activities, the share of rangeland dust sources has decreased by up to 85.6% compared to Niger (i.e. a decrease of 13.4%). In these countries, the share of cropland dust sources is 11.34%, 14.4%, 8.61%, and 7.89%, respectively (Fig. 6c). Accordingly, a high share of a LULC cannot necessarily be interpreted as a higher potential for dust emissions. Since croplands are highly exposed to land degradation processes due to unsustainable anthropogenic agricultural activities, and given the presence of soil particles that are more susceptible to aeolian processes^87^, they may have a much higher potential for dust emissions than rangelands. However, as a fact, our results suggest the dominant role of rangelands in global vegetative dust sources.

The area distribution of global desert dust sources (including sandy and non-sandy) in the countries involved is illustrated in Fig. 6d. Algeria, Libya, and Saudi Arabia are the top three countries in terms of desert dust sources with shares of 14.27%, 11.44%, and 10.94%, respectively. Together, these countries account for more than a third of the world’s desert dust sources. In all countries with desert dust sources, except Iran, the United States, Mexico, and Australia, sandy sources dominate. The largest share of non-sandy desert dust sources observed in China ($\:\sim$24%) and Sudan ($\:\sim$12%). Sand particles, typically between 0.05 and 2 mm in diameter, are coarser than other soil particles. Due to their greater weight, these particles are less likely to be detached from the surface by aeolian processes than clay and silt particles^9^. Assuming that main dust drivers such as soil moisture, wind speed and direction, and vegetation remain constant, sandy soils may have a lower dust emission potential compared to silt and clay. On the other hand, heavier particles have a lower mobility as a function of the wind speed and the force of gravity. In this regard, as acknowledged by Shepherd et al. (2016), most soil particles transported by the wind more than 100 km from their emission sources are smaller than 20 μm in diameter^88^. Accordingly, as a fact that requires further investigation, non-sandy dust sources, regardless of their extent, maybe more hazardous due to the greater potential for emission of fine-grained soil particles and affecting more distant areas.

### Contribution of natural and anthropogenic factors in the formation of global dust sources

The various types of dust sources identified in North Africa (Fig. 3), Southwest and Central Asia (Fig. 4), and East Asia (Fig. 5) generally result from the interaction between natural processes and human activities. However, it is important to note that making a precise distinction between the causes of dust source formation is challenging, as both natural and anthropogenic factors often contribute simultaneously and interactively on a global scale. However, as highlighted in the research literature^46,89^, desert dust sources are predominantly shaped by natural drivers, whereas non-desert sources—such as vegetative and hydrological areas—are primarily influenced by anthropogenic activities. Accordingly, it can be said that in desert areas, natural factors such as aeolian processes, hydroclimatic conditions (low rainfall, high temperature, low soil moisture, and high evaporation), and sparse vegetation cover contribute to the formation of dust sources. Regarding vegetative dust sources, the issue of LULC changes^90,91^ caused by human activities as well as climate change in the form of rangeland destruction and abandonment of agricultural lands play a role in dust emissions^1^. Hydrological dust sources, such as dried or ephemeral lakes and wetlands, are formed by natural hydroclimatic variability (e.g., drought periods) and human-induced upstream development such as dam construction, water diversions, and agricultural expansion^29,83^. As illustrated above, considering the area distribution of various global dust sources, the contribution of natural and anthropogenic factors can be estimated at 65% and 35%, respectively. These percentages may vary depending on the regional distribution of dust source types across North Africa, Southwest and Central Asia, and East Asia.

### Uncertainty and accuracy of the dust source maps

Considering the pixel size of the AAI product (spatial resolution of approximately 1 km and a detectable object dimension of around 2 km), the resulting thematic maps (Figs. 3, 4 and 5) have an estimated scale of approximately 1:2,000,000. In comparison, one of the most successful global dust maps by Ginoux et al. (2012) is at an approximate scale of 1:20,000,000, indicating that the maps produced in this study offer roughly a tenfold improvement in spatial detail^26^. Additionally, the aerosol load approach utilizing Sentinel 5P-AAI products, combined with a multi-stage masking process to exclude non-dust potential areas, constitutes an innovative aspect of this study that significantly reduces uncertainties in the identification of global dust sources. However, it is worth noting that the use of mean AAI data over a seven-year period (2018–2024) may lead to the exclusion of short-lived or episodic dust events. Temporary dust storms or seasonal variations that do not persist over multiple years could be averaged out, resulting in a loss of temporal detail. While this averaging approach is useful for identifying persistent dust sources, it may not fully account for the variability and intensity of dust emissions that occur on shorter time scales. Moreover, this study does not account for dust generated from non-traditional sources, such as those resulting from the melting of polar and mountain glaciers and permafrost^16^, which can release particulate matter previously trapped in ice layers.

The new approach used in this study aims to be simple, straightforward, generalizable, and quickly applicable on a global scale by leveraging the capabilities of UV-AAI and its optimal thresholding, which resulted in accurately identifying global dust sources. In this regard, the sources identified in the Middle East (Fig. 4) show a spatial agreement of over $\:\sim$80% with the ground truth dust areas (Fig. 2). It should be clarified that, the identified dust sources are based on an atmospheric aerosol loading approach and therefore may not necessarily be geometrically aligned with the ground truth dust areas, which are linked to specific landforms.

The spatial accuracy of the generated global dust source map was quantitatively evaluated using confusion matrix-based Precision, Recall, and F1-score metrics. This assessment was based on a comparison between the model’s output classes (dust sources and non-dust sources) with 3,000 randomly selected dust and non-dust hotspots as ground truth data obtained from visual interpretation across the Middle East. The model achieved a Precision of 0.847, indicating that a high proportion of areas identified as dust sources by the model corresponded correctly to dust hotspots. The Recall value of 0.807 reflects the model’s ability to successfully detect the majority of dust hotspots present in the ground truth, demonstrating its effectiveness in minimizing omissions. The F1-score of 0.826, as a harmonic mean of Precision and Recall, confirms the balanced and reliable performance of the proposed methodology in identifying spatial patterns of global dust source areas.

Previous global maps^12,25,26^ appear to have significantly overestimated the extent of identified dust sources due to significant uncertainty in the data and methodologies applied. In light of these limitations, we present the most recent and accurate global dust atlas, which is based on the mapping and characterization of satellite remote sensing data. While the methodology employed in this study is designed to be globally consistent, cost-effective, and broadly applicable, it does not explicitly account for regional ecological, climatic, and geomorphological variations that may influence dust emission processes. We acknowledge that variations in vegetation cover, soil characteristics, LULC, and hydroclimatic factors across regions can affect both the frequency and intensity of dust emissions. Future studies could enhance the methodology by integrating region-specific parameters or adaptive thresholds to better reflect local environmental conditions.

### Global dust calendar

**Fig. 7:**
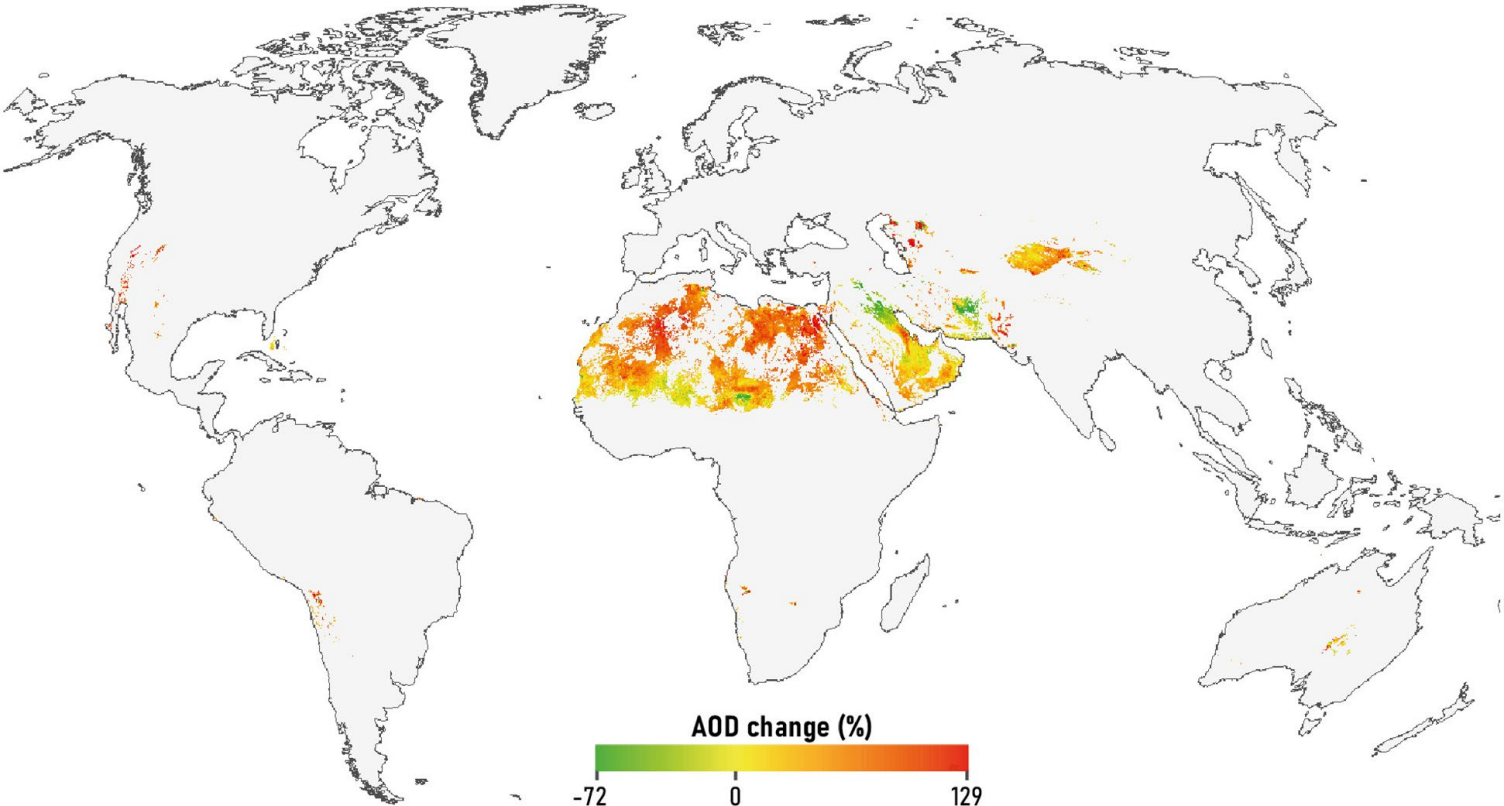
Detection of global MODIS-AOD change in dust sources from 2003–2013 to 2013–2024. The percentage of change in AOD is scaled from green (i.e., decrease in dust emission) to red (i.e., increase in dust emission).

**Fig. 8:**
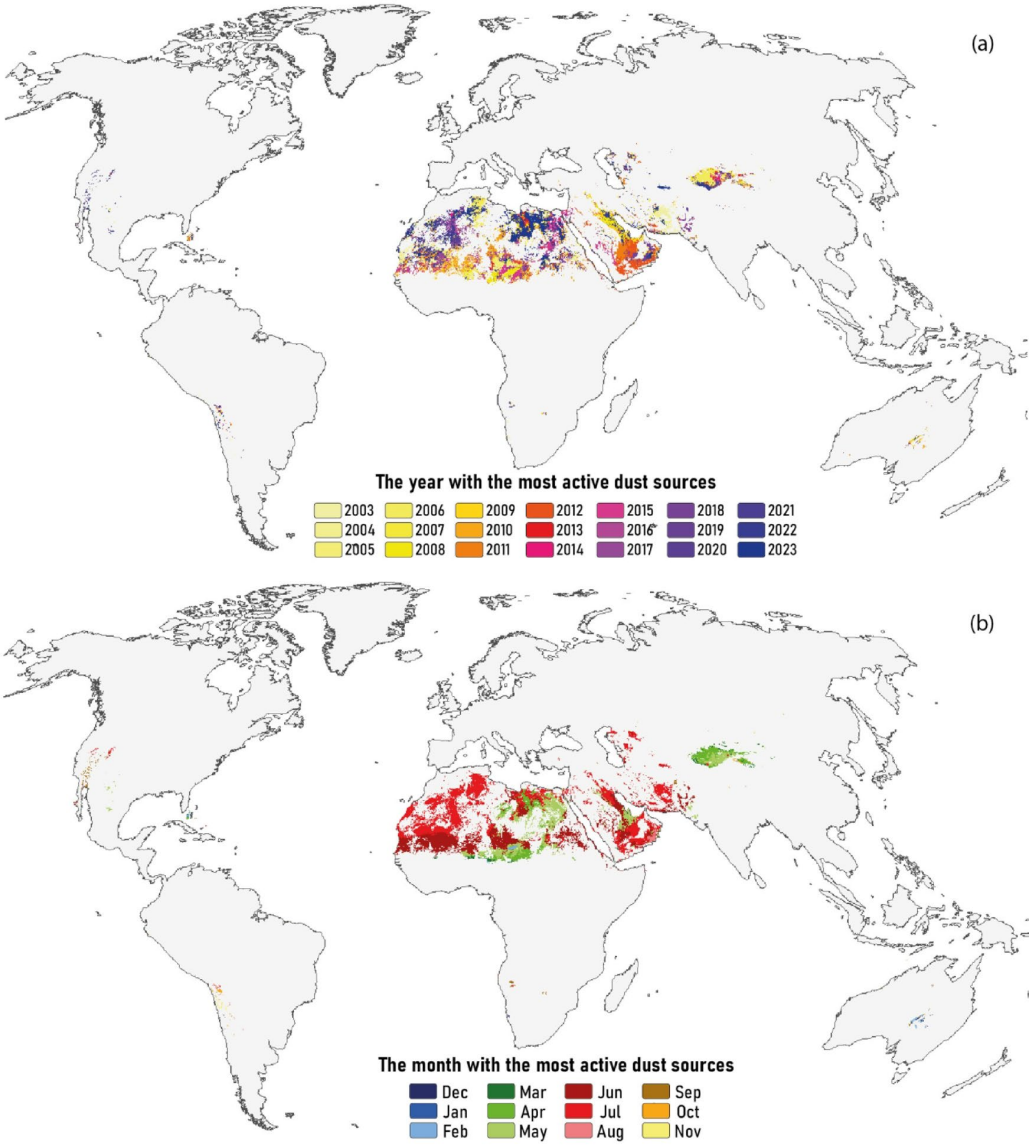
Global spatial distribution of the most active dust sources: (**a**) on an annual basis, and (**b**) on a monthly basis.

Comparing two distinct time periods, 2003–2013 and 2013–2024, the global trend in AOD associated with dust sources shows an overall increase (Fig. 7). This indicates that on a global scale, except for limited areas such as Mesopotamia and south-eastern Iran in southwest Asia, southern and south-western Niger, and southern and south-eastern Mali in North Africa, dust emissions have increased significantly over more than two decades. It is noteworthy that areas experiencing declines in dust activity largely overlap with vegetative and hydrological dust sources. Accordingly, the intensification of dust emissions has occurred more in desert areas, especially in North Africa and East Asia. The increasing frequency and intensity of dust storms in many parts of the world is largely attributed to factors such as LULC change, desertification, and the intensification of arid conditions driven by global climate change^92,93^. Contrasting the global trend, the decrease in dust emissions observed in the Middle East and southeastern Iran is closely linked to significant dust storm activity during the mid-2000s, particularly in 2007 and 2008. These periods of heightened activity were driven by prolonged droughts and human interventions^29^. This pattern of intense dust activity during those years is clearly illustrated in Fig. 8a.

Dust storms are driven by the interaction of various hydroclimatic and environmental conditions, resulting in distinct annual and seasonal dust patterns. Fig. 8a illustrates the annual distribution of dust activity across global sources, highlighting the years and regions with the highest emissions based on long-term MODIS-AOD data. The results reveal that dust sources in North Africa were particularly active during the second decade of the 21st century, whereas sources in Southwest and Central Asia, as well as East Asia, showed greater activity during the first decade (Fig. 8a).

Regarding monthly patterns, dust storms in the Northern Hemisphere are most prevalent in late spring and summer, especially from March to May and June to August (Fig. 8b). In East Asia, dust activity typically peaks during late winter to spring (March–May). Similar seasonal patterns are observed over large parts of Libya, Egypt, Sudan, Chad, and Niger in North Africa. In the Southern Hemisphere, the highest dust storm activity generally occurs between September and November, aligning with late winter and spring months, particularly in regions such as Australia and South America.

## Conclusion

This study identified high-resolution (0.01°) global dust sources using multitemporal satellite remote sensing data. An aerosol loading approach based on the Sentinel-5P absorbing aerosol index (AAI) was employed to characterize dust sources. We produced a global dust atlas to provide an up-to-date understanding of this destructive environmental phenomenon.

First, a global mean AAI map (2018–2024) was generated. Using a histogram analysis method based on ground truth dust emission areas across the Middle East–obtained through visual interpretation of MODIS true-color composite imagery–an average AAI threshold of 0.25 was established to identify potential global dust sources. By excluding areas with no dust potential from the thresholded mean AAI map, an accurate global dust source map was produced at a scale of approximately 1:2,000,000. The validation results, with a Precision of 84.7%, Recall of 80.7%, and F1-score of 82.6%, confirm the proposed method’s strong spatial accuracy and reliability in identifying global dust source areas. Additionally, spatial overlap analysis with different land use/cover (LULC) types was employed to categorize dust sources into three classes–desert, vegetative, and hydrological–each further divided into specific subclasses.

Our findings indicate that North Africa accounts for the largest proportion of global dust sources (67%), followed by Asia—particularly the Southwest, Central, and East regions—which contributes approximately 30%. Scattered and low-extent dust sources were also identified in other regions, including North and South America, Australia, and Europe. In terms of source types, deserts contribute around 65%, vegetative sources 26%, and hydrological sources 9%. While the distribution of these source types varies by region, desert sources are the predominant type globally. In North Africa, Southwest Asia, and Central Asia, the distribution of dust source types aligns with global trends. However, in East Asia, hydrological dust sources account for a greater share than vegetative sources. Among global vegetative dust sources, rangelands contribute the most, accounting for over 89%, compared to croplands. Intermittent water bodies (20–56%) and floodplains (21–34%) represent the primary hydrological dust sources across many regions worldwide. Our analysis also reveals the contributions of individual countries to different dust source types globally. Algeria, Libya, and Saudi Arabia are the top three contributors to desert dust sources, collectively accounting for more than one-third of the global total. Niger holds the largest area of vegetative dust sources, with rangelands comprising approximately 99% of this category. China is the leading contributor to global hydrological dust sources, with over 50% of these associated with intermittent water bodies.

An examination of the global distribution of dust sources and the underlying causes of their formation indicates that both natural and anthropogenic factors interact to drive dust emissions across different regions. Accurately quantifying the individual contributions of these factors remains complex and challenging. However, desert dust sources are generally associated with natural processes, whereas non-desert sources–such as vegetative and hydrological types–are predominantly influenced by human activities. Overall, natural and anthropogenic factors are estimated to contribute approximately 65% and 35%, respectively, to the formation of global dust sources.

Analysis of global dust patterns reveals an overall increase in emissions from desert sources in recent decades. However, certain regions, particularly the Middle East, exhibit a reverse trend, with rising emissions from vegetative and hydrological dust sources. This shift is largely attributed to intensified dust activity during severe droughts in the early 21st century. The annual pattern of dust events shows that dust sources in North Africa were most active during the second decade of this century, while those in Southwest, Central, and East Asia peaked during the first decade. Across both regions, dust emissions from primary sources occur most frequently in summer, with a secondary peak in late winter to spring.

This study presents a novel and effective global dust source mapping methodology by leveraging the Sentinel-5P AAI product combined with a multi-stage spatial filtering approach. This strategy greatly enhances the spatial precision and reliability of identifying dust sources, making it a valuable tool for climate modeling, air quality forecasting, and environmental risk assessment. The AAI product offers daily temporal resolution; however, for the purpose of this study, it was averaged over the period from 2018 to 2024 to generate the primary potential dust source map. As a result, short-lived or episodic dust events may not be fully captured in the final analysis. Accordingly, the proposed methodology focuses on identifying stable dust-emitting sources by considering areas with relatively high aerosol load over time.

To build upon the findings of this study, future research could integrate higher temporal resolution analyses by incorporating shorter time windows or seasonal aggregations of the AAI data to better capture transient or episodic dust events. Additionally, combining high spatial resolution Sentinel-5P AAI data with complementary satellite products (e.g., Suomi NPP-VIIRS, Meteosat-SEVIRI, or CALIPSO) and ground-based observations may further improve the temporal sensitivity and vertical characterization of dust emissions. Incorporating meteorological parameters such as wind speed, soil moisture, and surface roughness could also enhance the dynamic modeling of dust mobilization processes. Moreover, the application of machine learning or data assimilation techniques could provide a more adaptive and predictive framework for identifying emerging or shifting dust source areas under changing climatic conditions.

## Data Availability

The datasets used and/or analyzed during the current study available from the corresponding author on reasonable request.

## References

[1] Darvishi Boloorani, A. et al. Assessing the role of drought in dust storm formation in the Tigris and euphrates basin. *Sci. Total Environ.* **921**, 171193. 10.1016/j.scitotenv.2024.171193 (2024). 38402961 10.1016/j.scitotenv.2024.171193

[2] Zittis, G. et al. Climate change and weather extremes in the Eastern Mediterranean and Middle East. *Rev. Geophys.* **60**, e2021RG000762 (2022).

[3] Chiang, F., Mazdiyasni, O. & AghaKouchak, A. Evidence of anthropogenic impacts on global drought frequency, duration, and intensity. *Nat. Commun.* **12**(1), 2754 (2021). 33980822 10.1038/s41467-021-22314-w PMC8115225

[4] Trenberth, K. E. et al. Global warming and changes in drought. *Nat. Clim. Change* **4**(1), 17–22 (2014).

[5] Trenberth, K. E. Climate change caused by human activities is happening and it already has major consequences. *J. Energy Nat. Resour. Law* **36**(4), 463–481 (2018).

[6] Stern, D. I. & Kaufmann, R. K. Anthropogenic and natural causes of climate change. *Clim. Change* **122**, 257–269 (2014).

[7] Middleton, N. Impacts of sand and dust storms on food production. *Environ. Res. Food Syst.* **1**(2), 22003 (2024).

[8] Darvishi Boloorani, A. et al. In *Microbiology of Sand and Dust Storms and the Effects on Human Health in Iran and Other Persian Gulf Countries BT - Dust and Health: Challenges and Solutions* 157–186 (eds Al-Dousari, A. & Hashmi, M. Z.) (Springer International Publishing, 2023). 10.1007/978-3-031-21209-3_9

[9] Darvishi Boloorani, A. et al. In *Sources, Drivers, and Impacts of Sand and Dust Storms: A Global View BT - Dust and Health: Challenges and Solutions* 31–49 (eds Al-Dousari, A. & Hashmi, M. Z.) (Springer International Publishing, 2023). 10.1007/978-3-031-21209-3_3.

[10] Kok, J. F. et al. Mineral dust aerosol impacts on global climate and climate change. *Nat. Rev. Earth Environ.* **4**(2), 71–86 (2023).

[11] Darvishi Boloorani, A. et al. A new approach to dust source mapping using visual interpretation and object-oriented segmentation of satellite imagery. *Appl. Comput. Geosci.* **23**, 100182. 10.1016/j.acags.2024.100182 (2024).

[12] Kim, D. et al. Where dust comes from: Global assessment of dust source attributions with Aerocom models. *J. Geophys. Res. Atmos.* **129**, e2024JD041377 (2024).

[13] Shao, Y. et al. Dust cycle: An emerging core theme in Earth system science. *Aeolian Res.* **2**(4), 181–204 (2011).

[14] Wang, J. X. L. Mapping the global dust storm records: Review of dust data sources in supporting modeling/climate study. *Curr. Pollut. Rep.* **1**(2), 82–94 (2015).

[15] Kim, D. et al. Sources, sinks, and transatlantic transport of North African dust aerosol: A multimodel analysis and comparison with remote sensing data. *J. Geophys. Res. Atmos.* **119**(10), 6259–6277 (2014).

[16] Bullard, J. E. et al. High-latitude dust in the Earth system. *Rev. Geophys.* **54**(2), 447–485 (2016).

[17] Al-Hemoud, A. et al. Dust source susceptibility in the lower Mesopotamian floodplain of Iraq. *Remote Sens. Appl. Soc. Environ.* **36**, 101355 (2024).

[18] Chen, W., Meng, H., Song, H. & Zheng, H. Progress in dust modelling, global dust budgets, and soil organic carbon dynamics. *Land*. 10.3390/land11020176 (2022).

[19] Zender, C. S., Newman, D. & Torres, O. Spatial heterogeneity in aeolian erodibility: Uniform, topographic, geomorphic, and hydrologic hypotheses. *J. Geophys. Res. Atmos.* **108**, D17 (2003).

[20] Wang, N. & Zhang, Y. Long-term variations of global dust emissions and climate control. *Environ. Pollut.* **340**, 122847. 10.1016/j.envpol.2023.122847 (2024). 37918770 10.1016/j.envpol.2023.122847

[21] Tegen, I. & Fung, I. Contribution to the atmospheric mineral aerosol load from land surface modification. *J. Geophys. Res. Atmos.* **100**(D9), 18707–18726 (1995).

[22] Ginoux, P. et al. Sources and distributions of dust aerosols simulated with the GOCART model. *J. Geophys. Res. Atmos.* **106**, 20255–20273 (2001).

[23] Herman, J. R. et al. Global distribution of UV-absorbing aerosols from nimbus 7/TOMS data. *J. Geophys. Res. Atmos.* **102**(D14), 16911–16922 (1997).

[24] Washington, R., Todd, M., Middleton, N. J. & Goudie, A. S. Dust-storm source areas determined by the total Ozone monitoring spectrometer and surface observations. *Ann. Assoc. Am. Geogr.* **93**(2), 297–313 (2003).

[25] Prospero, J. M., Ginoux, P., Torres, O., Nicholson, S. E. & Gill, T. E. Environmental characterization of global sources of atmospheric soil dust identified with the nimbus 7 total Ozone mapping spectrometer (TOMS) absorbing aerosol product. *Rev. Geophys.* **40**(1), 1–2 (2002).

[26] Ginoux, P., Prospero, J. M., Gill, T. E., Hsu, N. C. & Zhao, M. Global-scale attribution of anthropogenic and natural dust sources and their emission rates based on MODIS deep blue aerosol products. *Rev. Geophys.*10.1029/2012RG000388 (2012).

[27] Rayegani, B. et al. Sand and dust storm sources identification: A remote sensing approach. *Ecol. Indic.* **112**, 106099 (2020).

[28] Baddock, M. C., Bryant, R. G., Acosta, M. D. & Gill, T. E. Understanding dust sources through remote sensing: Making a case for cubesats. *J. Arid Environ.* **184**, 104335. 10.1016/j.jaridenv.2020.104335 (2021).

[29] Darvishi Boloorani, A. et al. Water bodies changes in Tigris and euphrates basin has impacted dust storms phenomena. *Aeolian Res.* **50**, 100698 (2021).

[30] Von Holdt, J. R., Eckardt, F. D. & Wiggs, G. F. S. Landsat identifies aeolian dust emission dynamics at the landform scale. *Remote Sens. Environ.* **198**, 229–243 (2017).

[31] O’Loingsigh, T. et al. Correction of dust event frequency from MODIS Quick-Look imagery using in-situ aerosol measurements over the lake Eyre basin, Australia. *Remote Sens. Environ.* **169**, 222–231 (2015).

[32] Sinclair, S. N. & LeGrand, S. L. Reproducibility assessment and uncertainty quantification in subjective dust source mapping. *Aeolian Res.* **40**, 42–52 (2019).

[33] Darvishi Boloorani, A. et al. Visual interpretation of satellite imagery for hotspot dust sources identification. *Remote Sens. Appl. Soc. Environ.* **29**, 100888 (2023).

[34] Kok, J. F. et al. Contribution of the world’s main dust source regions to the global cycle of desert dust. *Atmos. Chem. Phys.* **21**(10), 8169–8193 (2021).

[35] Hsu, N. C., Tsay, S. C., King, M. D. & Herman, J. R. Aerosol properties over bright-reflecting source regions. *IEEE Trans. Geosci. Remote Sens.* **42**(3), 557–569 (2004).

[36] Hsu, N. C., Tsay, S. C., King, M. D. & Herman, J. R. Deep blue retrievals of Asian aerosol properties during ACE-Asia. *IEEE Trans. Geosci. Remote Sens.* **44**(11), 3180–3195 (2006).

[37] Levy, R. C. et al. The collection 6 MODIS aerosol products over land and ocean. *Atmos. Meas. Tech.* **6**(11), 2989–3034 (2013).

[38] Soleimani, M., Argany, M., Papi, R. & Amiri, F. Satellite aerosol optical depth prediction using data mining of climate parameters. *Phys. Geogr. Res. Q.* **53**(3), 319–333. 10.22059/jphgr.2021.318600.1007591 (2021).

[39] Papi, R. et al. Identifying sand and dust storm sources using spatial-temporal analysis of remote sensing data in central Iran. *Ecol. Inf.* **70**, 101724 (2022).

[40] Darvishi Boloorani, A. et al. Assessment of rural vulnerability to sand and dust storms in Iran. *Atmosphere*. 10.3390/atmos14020281 (2023).

[41] Sharma, V., Ghosh, S., Bilal, M., Dey, S. & Singh, S. Performance of MODIS C6.1 dark target and deep blue aerosol products in Delhi National capital region, India: Application for aerosol studies. *Atmos. Pollut. Res.* **12**(3), 65–74. 10.1016/j.apr.2021.01.023 (2021).

[42] Eibedingil, I. G., Gill, T. E., Van Pelt, R. S. & Tong, D. Q. Comparison of aerosol optical depth from MODIS product collection 6.1 and AERONET in the Western United States. *Remote Sens.*10.3390/rs13122316 (2021).

[43] Chen, Q. X. et al. Evaluation of MODIS, MISR, and VIIRS daily level-3 aerosol optical depth products over land. *Atmos. Res.* **265**, 105810 (2022).

[44] Wei, J., Li, Z., Peng, Y. & Sun, L. MODIS collection 6.1 aerosol optical depth products over land and ocean: Validation and comparison. *Atmos. Environ.* **201**, 428–440 (2019).

[45] Darvishi Boloorani, A., Neysani Samany, N., Papi, R. & Soleimani, M. Dust source susceptibility mapping in Tigris and euphrates basin using remotely sensed imagery. *Catena* **209**, 105795 (2022).

[46] Papi, R., Attarchi, S., Darvishi Boloorani, A. & Neysani Samany, N. Characterization of hydrologic sand and dust storm sources in the middle East. *Sustainability* **14**(22), 15352 (2022).

[47] Papi, R., Argany, M., Moradipour, S. & Soleimani, M. Modeling the potential of sand and dust storm sources formation using time series of remote sensing data, fuzzy logic and artificial neural network (A case study of euphrates basin). *J. Geospatial Inf. Technol.*10.52547/jgit.8.3.61 (2021).

[48] Papi, R., Attarchi, S., Boloorani, A. D. & Samany, N. N. Knowledge discovery of middle East dust sources using apriori Spatial data mining algorithm. *Ecol. Inf.* **72**, 101867 (2022).

[49] Pelletier, J. D. et al. Global 1-km gridded thickness of soil, regolith, and sedimentary deposit layers. *ORNL DAAC* (2016).

[50] Pekel, J. F., Cottam, A., Gorelick, N. & Belward, A. S. High-resolution mapping of global surface water and its long-term changes. *Nature* **540**(7633), 418–422 (2016). 27926733 10.1038/nature20584

[51] Karra, K. et al. Global land use/land cover with Sentinel 2 and deep learning. In *2021 IEEE International Geoscience and Remote Sensing Symposium IGARSS* 4704–4707 (2021).

[52] Sokolova, M. & Lapalme, G. A systematic analysis of performance measures for classification tasks. *Inf. Process. Manag.* **45**(4), 427–437 (2009).

[53] Powers, D. M. W. Evaluation: From precision, recall and F-measure to ROC, informedness, markedness and correlation. arXiv prepint https://arxiv.org/abs/2010.16061 (2010).

[54] Hengl, T. Soil texture classes (USDA system) for 6 soil depths (0, 10, 30, 60, 100 and 200 cm) at 250 m. *Zenodo Ed* (2018).

[55] Farr, T. G. et al. The shuttle radar topography mission. *Rev. Geophys.* **45**(2) (2007).

[56] Neumann, J. V. Distribution of the ratio of the mean square successive difference to the variance. *Ann. Math. Stat.* **12**(4), 367–395 (1941).

[57] Pettitt, A. N. A non-parametric approach to the change‐point problem. *J. R. Stat. Soc. Ser. C Appl. Stat.* **28**(2), 126–135 (1979).

[58] Khaliq, M. N. & Ouarda, T. B. M. J. On the critical values of the standard normal homogeneity test (SNHT). *Int. J. Climatol. J. R Meteorol. Soc.* **27**(5), 681–687 (2007).

[59] Alexandersson, H. A homogeneity test applied to precipitation data. *J. Climatol.* **6**(6), 661–675. 10.1002/joc.3370060607 (1986).

[60] Buishand, T. A. Some methods for testing the homogeneity of rainfall records. *J. Hydrol.* **58**, 1–2 (1982).

[61] Buishand, T. A. Tests for detecting a shift in the mean of hydrological time series. *J. Hydrol.* **73**, 1–2 (1984).

[62] Rashki, A., Middleton, N. J. & Goudie, A. S. Dust storms in Iran–Distribution, causes, frequencies and impacts. *Aeolian Res.* **48**, 100655 (2021).

[63] Nobakht, M., Shahgedanova, M. & White, K. New inventory of dust emission sources in central Asia and Northwestern China derived from MODIS imagery using dust enhancement technique. *J. Geophys. Res. Atmos.* **126**, e2020JD033382 (2021).

[64] Scheuvens, D., Schütz, L., Kandler, K., Ebert, M. & Weinbruch, S. Bulk composition of Northern African dust and its source sediments—A compilation. *Earth Sci. Rev.* **116**, 170–194. 10.1016/j.earscirev.2012.08.005 (2013).

[65] Zhang, C., Gao, R., Wu, J. & Yang, Z.* Combating Climate Change, Desertification and Sandstorms: A Collaborative Approach BT - Annual Report on China’s Response to Climate Change: Implementing The Paris Agreement* 145–153 (eds Wang, W. & Liu, Y.) (Springer Nature Singapore, 2020). 10.1007/978-981-13-9660-1_13.

[66] Schepanski, K., Mallet, M., Heinold, B. & Ulrich, M. North African dust transport toward the Western mediterranean basin: Atmospheric controls on dust source activation and transport pathways during June–July 2013. *Atmos. Chem. Phys.* **16**(22), 14147–14168 (2016).

[67] Schepanski, K., Heinold, B. & Tegen, I. Harmattan, saharan heat low, and West African monsoon circulation: modulations on the saharan dust outflow towards the North Atlantic. *Atmos. Chem. Phys.* **17**(17), 10223–10243 (2017).

[68] Prospero, J. M., Collard, F., Molinié, J. & Jeannot, A. Characterizing the annual cycle of African dust transport to the Caribbean basin and South America and its impact on the environment and air quality. *Glob. Biogeochem. Cycles* **28**(7), 757–773 (2014).

[69] Liu, Q., Huang, Z., Hu, Z., Dong, Q. & Li, S. Long-range transport and evolution of saharan dust over East Asia from 2007 to 2020. *J. Geophys. Res. Atmos.* **127**, e2022JD036974 (2022).

[70] Gandham, H., Dasari, H. P., Saharwardi, M. S., Karumuri, A. & Hoteit, I. Dust sources over the Arabian Peninsula. *Environ. Res. Lett.* **18**(9), 94053 (2023).

[71] Mousavi, H., Panahi, D. M. & Kalantari, Z. Dust and climate interactions in the middle east: Spatio-temporal analysis of aerosol optical depth and Climatic variables. *Sci. Total Environ.* **927**, 172176. 10.1016/j.scitotenv.2024.172176 (2024). 38575026 10.1016/j.scitotenv.2024.172176

[72] Rashki, A. et al. Effects of monsoon, Shamal and Levar winds on dust accumulation over the Arabian sea during summer—The July 2016 case. *Aeolian Res.* **36**, 27–44. 10.1016/j.aeolia.2018.11.002 (2019).

[73] Alizadeh-Choobari, O., Zawar-Reza, P. & Sturman, A. The ‘wind of 120days’ and dust storm activity over the Sistan basin. *Atmos. Res.* **143**, 328–341. 10.1016/j.atmosres.2014.02.001 (2014).

[74] Sun, J. et al. Extreme aridification since the beginning of the pliocene in the Tarim basin, Western China. *Palaeogeogr. Palaeoclimatol. Palaeoecol.* **485**, 189–200. 10.1016/j.palaeo.2017.06.012 (2017).

[75] Hu, T. et al. Morphology and mineralogical composition of sandblasting dust particles from the Taklimakan desert. *Sci. Total Environ.* **834**, 155315. 10.1016/j.scitotenv.2022.155315 (2022). 35447171 10.1016/j.scitotenv.2022.155315

[76] Wang, H., Jia, X., Li, K. & Wang, H. External supply of dust in the Taklamakan sand sea, Northwest china, reveals the dust-forming processes of the modern sand sea surface. *Catena* **119**, 104–115. 10.1016/j.catena.2014.03.015 (2014).

[77] Zhang, J. et al. Prolonged drought enhances Northwest China dust storm activity. *J. Geophys. Res. Atmos.* **127**, e2022JD037088 (2022).

[78] Zhang, X. et al. Identification of dust sources and hotspots in East Asia during 2000–2015: Implications for numerical modeling and forecasting. *Atmos. Chem. Phys. Discuss.* **2016**, 1–42 (2016).

[79] Zu, R. et al. Characteristics of near-surface wind regimes in the Taklimakan desert, China. *Geomorphology* **96**(1), 39–47. 10.1016/j.geomorph.2007.07.008 (2008).

[80] Darvishi Boloorani, A. et al. In *Climate Change, Drought, and Water Scarcity in the MENA Region BT - Climate Change and Environmental Degradation in the MENA Region* 189–204 (eds Al-Quraishi, A., Negm, A. & Benzougagh, B.) (Springer Nature Switzerland, 2024). 10.1007/698_2024_1143.

[81] Ahmadi, H., Mousavi, B. S., Argany, M., Soleimani, M. & Ghanbari, A. Spatiotemporal forecasting of water change trends in urmia lake through to 2030, using STC-based models. *Hydrol. Sci. J.* 1–21 (2024).

[82] Boroughani, M., Hashemi, H., Hosseini, S. H., Pourhashemi, S. & Berndtsson, R. Desiccating lake urmia: A new dust source of regional importance. *IEEE Geosci. Remote Sens. Lett.* **17**(9), 1483–1487. 10.1109/LGRS.2019.2949132 (2020).

[83] Akbari, M. et al. Desiccation of the transboundary Hamun lakes between Iran and Afghanistan in response to Hydro-climatic droughts and anthropogenic activities. *J. Great Lakes Res.* **48**(4), 876–889. 10.1016/j.jglr.2022.05.004 (2022).

[84] Darvishi Boloorani, A., Najafi, M. S., Soleimani, M., Papi, R. & Torabi, O. Influence of Hamoun lakes’ dry conditions on dust emission and radiative forcing over Sistan plain, Iran. *Atmos. Res.* **272**, 106152 (2022).

[85] Banks, J. R., Heinold, B. & Schepanski, K. Impacts of the desiccation of the Aral sea on the central Asian dust life-cycle. *J. Geophys. Res. Atmos.* **127**, e2022JD036618 (2022).

[86] Wang, W. et al. Temporal characterization of sand and dust storm activity and its Climatic and terrestrial drivers in the Aral sea region. *Atmos. Res.* **275**, 106242. 10.1016/j.atmosres.2022.106242 (2022).

[87] Darvishi Boloorani, A. et al. Land degradability mapping using remote sensing data and soil chemical properties. *Remote Sens. Appl. Soc. Environ.* **32**, 101027 (2023).

[88] Shepherd, G. et al. Global assessment of sand and dust storms (2016).

[89] Darvishi Boloorani, A. et al. In *Climate Change, Dust Storms, and Air Pollution in the MENA Region BT - Climate Change and Environmental Degradation in the MENA Region* 327–343 (eds Al-Quraishi, A., Negm, A. & Benzougagh, B.) (Springer Nature Switzerland, 2024). 10.1007/698_2024_1144.

[90] Hashemi, H. et al. Unraveling the link between agricultural patterns and dust storm occurrence in Mesopotamia. *Environ. Res. Lett.* (2025).

[91] Moridnejad, A., Karimi, N. & Ariya, P. A. Newly desertified regions in Iraq and its surrounding areas: Significant novel sources of global dust particles. *J. Arid Environ.* **116**, 1–10 (2015).

[92] Xi, X. Global aeolian dust variations and trends: A revisit of dust event and visibility observations from surface weather stations. *Atmos. Chem. Phys. Discuss.* **2020**, 1–34 (2020).

[93] Goudie, A. S. & Middleton, N. J. The changing frequency of dust storms through time. *Clim. Change* **20**(3), 197–225. 10.1007/BF00139839 (1992).

